# LncRNA SNHG7 Regulates Mesenchymal Stem Cell Through the Notch1/Jagged1/Hes-1 Signaling Pathway and Influences Folfirinox Resistance in Pancreatic Cancer

**DOI:** 10.3389/fonc.2021.719855

**Published:** 2021-09-22

**Authors:** Dongfeng Cheng, Juanjuan Fan, Kai Qin, Yiran Zhou, Jingrui Yang, Yang Ma, Minmin Shi, Jiabin Jin

**Affiliations:** ^1^ Pancreatic Disease Center, Department of General Surgery, Rui Jin Hospital Affiliated to Shanghai Jiao Tong University School of Medicine, Shanghai, China; ^2^ Department of General Surgery, Yichuan Community Health, Shanghai, China; ^3^ Department of Surgery, Ruijin Hospital, Shanghai Jiaotong University School of Medicine, Shanghai, China; ^4^ Research Institute of Digestive Surgery, Ruijin Hospital, Shanghai Jiaotong University School of Medicine, Shanghai, China

**Keywords:** SNHG7, Notch1, pancreatic cancer, stemness, resistance

## Abstract

Pancreatic cancer (PC) is one of the deadliest gastrointestinal cancers, accounting for the fourth highest number of cancer-related fatalities. Increasing data suggests that mesenchymal stem cells (MSCs) might influence the drug resistance of GC cells in the tumor microenvironment and play essential roles in drug resistance development. However, the precise underlying process remains a mystery. The purpose of this study was to look at the control of MSC-induced SNHG7 in pancreatic cancer. *In vitro* and *in vivo* sphere formation, colony formation, and flow cytometry investigations revealed the stemness and Folfirinox resistance in pancreatic cancer cells. To confirm the direct connections between SNHG7 and other related targets, RNA pulldown and immunoprecipitation tests were performed. MSC co-culture enhanced the stemness and Folfirinox resistance in pancreatic cancer cells according to the findings. MSC co-culture increased SNHG7 expression in pancreatic cancer cells, contributing to the stemness and Folfirinox resistance. We demonstrated that Notch1 interacted with SNHG7 and could reverse the facilitative effect of SNHG7 on the stemness and Folfirinox resistance in pancreatic cancer cells. Finally, our findings showed that MSCs increased SNHG7 expression in pancreatic cancer cells, promoting the stemness and Folfirinox resistance *via* the Notch1/Jagged1/Hes-1 signaling pathway. These findings could provide a novel approach and therapeutic target for pancreatic cancer patients.

## Introduction

Pancreatic cancer is one of the deadliest gastrointestinal malignancies, accounting for the fourth highest number of cancer-related deaths (1). Pancreatic cancer develops at a rapid, unique, and accelerated rate (2). There is no clinically sensitive early diagnosis indication or effective treatment point as a result of a biological process encompassing numerous phases (3). As a result, the quest for diagnostic indicators and tailored medicines to slow the growth of pancreatic cancer has become a major emphasis in pancreatic cancer treatment.

It has been shown that just 2% of the genome sequence is capable of coding proteins, whereas non-coding RNA accounts for more than 95% of the transcripts (4). Long non-coding RNAs (lncRNAs) are a kind of non-coding RNA that is longer than 200 nucleotides and lacks the ability to code for proteins (nt). Emerging data suggests that lncRNA has a role in a variety of malignant tumors, including pancreatic cancer (5–7). For example, the lncRNA HOTTIP promotes pancreatic cancer by enhancing the Wnt/-catenin pathway by binding to WDR5 (8). Recently, there has been a progressive discovery in the investigation of small nucleolar RNA host gene 7 (SNGH7) in ovarian cancer (9). However, the precise functions of SNGH7 in pancreatic cancer are yet unknown. Most patients with pancreatic cancer can now be fully resected as far as feasible because of the advances in surgery and medication therapy in the recent years. However, many patients still experience recurrence or metastasis after surgical resection, with a poor prognosis. It is not difficult to find out the reasons for this. In addition to the highly malignant characteristics of pancreatic cancer itself, chemotherapy resistance is also an important reason. Folfirinox regimen is a common combination chemotherapy regimen for pancreatic cancer based on fluurazepine, and drug resistance often directly affects the prognosis of patients. Therefore, finding a solution to the drug resistance of Folfirinox regimen is an urgent need to explore (10).

In the present study, we investigated the functional role and regulatory mechanism of SNGH7 in the stemness and Folfirinox resistance of pancreatic cancer cells. We found that SNGH7 was induced under MSC-culture in pancreatic cancer and elevated SNGH7 promoted the stemness and Folfirinox resistance. Mechanistic investigations revealed that SNGH7 interacted with Notch1 to regulate the stemness and Folfirinox resistance through the Notch1/Jagged1/Hes-1 signaling pathway in pancreatic cancer.

## Materials and Methods

### Patients

Pancreatic cancer tissues (n = 50) were obtained by surgery and normal tissues adjacent to cancer were obtained at the same time from Ruijin Hospital Affiliated to Medical College of Shanghai Jiaotong University. All patients have signed informed consents. The study was approved by the ethics committee of Ruijin Hospital Affiliated to Medical College of Shanghai Jiaotong University and conducted under its supervision.

### Cell Culture

Human pancreatic cancer cells (PANC-1 and AsPC-1) and adult bone marrow MSCs were obtained from the Cell Resource Center of Shanghai Academy of Sciences. Cells were cultured in RPMI-1640 (Procell Life Science&Technology, Wuhan, China) with 10% fetal bovine (Thermo-Scientific, MA, USA) and 1% penicillin-streptomycin (MP Biomedicals, CA, USA). The cells were cultured in 5% CO2 and 37°C incubators. A Transwell cell culture room (Thermo, USA) was used for co-cultivation. In the co-culture system, MSCs were placed in the upper chamber and pancreatic cancer cells were placed in the lower chamber, allowing direct contact between MSCs and PC cells. Folfirinox is a common plan for pancreatic cancer chemotherapy to treat cells. It consists of four drugs, FOL-Folinicacid (CSNpharm, Shanghai, China), F-Fluorouracil (CSNpharm, Shanghai, China), IRIN-Irinotecan (CSNpharm, Shanghai), China) and OX-oxaliplatin (CSNpharm, Shanghai, China).

### Sphere-Forming Assay

Used 1× stem cell culture medium to adjust the cell density to 2 × 10^4^/mL, inoculated 500 microliters of cells per well in a 24-well ultra-low adsorption plate (Corning company, cat No. 3473), cultured at 37°C, 5% CO2, respectively. Added 10× stem cell culture medium (50µL/well) to culture for 3, 5, and 7 days. Collected cells after culture for 8 days. Centrifuged at 100×g for 2 min. Discarded the supernatant. Resuspended the spheroid cells in 200&microlL trypsin digestion solution and incubated at 37°C. In 3 minutes, added 800µL of serum-containing medium, and counted live cells using Countstar automatic cell counter (Alite, China). The formula of 1× stem cell culture medium was serum-free DMEM/F12 medium (Thermo, USA), containing 20 ng/mL EGF (Thermo, USA), 20 ng/mL bFGF (Thermo, USA), 4 µg/mL heparin (XiYa reagent company, China) and 1×B27 (Thermo, USA).

### Cell Transfection

When cells reached 60%–80% confluence, the transfection was performed. The SNHG7 vector and the control vector (GenePharma, Shanghai, China) were transfected using the Lipofectamine 2000 Reagent (Thermo-Scientific, MA, USA). After culturing for 48 h, cells were utilized for the follow-up study.

### Real-Time Quantitative Polymerase Chain Reaction (RT-qPCR)

Total RNAs were obtained from tissues and cells using TRIzol (Invitrogen, MA, USA). Reverse Transcription Kit (D1802, Haigene, Harbin, China) was used for the reverse transcription of RNA and obtain the cDNA. SYBR green PCR Kit (Vazyme, Nanjing, China) was used for RT-qPCR. U6 and GAPDH were used as the endogenous genes to normalize the relative expression of miRNAs and genes. Several studies have shown that the expression of SOX2, OCT-4, LIN28, and CD133 can be used as a marker for pancreatic cancer MSC, so we chose to detect the related expression of these four genes to reflect the change of stem cell characteristics (11).

### Western Blot

RIPA lysis buffer with 1 mM PMSF was used to extract the total protein (Solarbio, Beijing, China). BCA protein assay kit (Thermo Fisher, MA, USA) was used to measure the protein concentration after protein extraction. A total of 30 g of total proteins was separated using 10% SDS-PAGE gels and transferred to a PVDF membrane (Millipore, USA). After 2 h of blocking with 5% skim milk, the membrane was incubated overnight at 4°C with a particular primary antibody. The particular primary antibodies were bought from Abcam, and the concentration used in this investigation was 1:1,000. The membrane was then treated for 2 h at room temperature with the matching secondary antibodies. The protein signals are seen using the ECL Western blotting substrate (Tanon, Shanghai, China). The internal gene to indicate the relative expression was GAPDH.

### Colony Formation Assay

Cells were inoculated into a 6-well plate and the density was 500 cells/well. Then, the cells were cultured for 14 days in the appropriate medium. Following, the cells were fixed with methanol, washed twice with PBS, and stained with 0.1% crystal violet solution (Beyotime, Shanghai, China). The colonies were observed using a microscope (Nikon, Tokyo, Japan) and counted in six different fields.

### Flow Cytometry

Cells were collected and passed through 100-mesh sieves. Then, the cells were incubated for 15 min with the Annexin V and PI solution at room temperature. The staining process needed to avoid light. The labeled cells were analyzed through the FACS flow cytometry (Leica, Wetzlar, Germany).

### RNA Pulldown Assay

The pulldown assay was performed according to the previously described protocol (8). In brief, biotinylated miR-526b-3p wild type and biotinylated miR-526b-3p mutant or control probe were respectively transfected in PANC-1 and AsPC-1 cells. These probes were purchased from RiboBio (Guangzhou, China). A total of 10^7^ PANC-1 and AsPC-1 cells were harvested and lysed by a lysis buffer. The total RNA solution was added DNaseI and incubated for 5 min at 65°C, followed by an instant ice bath. The solution was then incubated for 4 h with streptavidin-coated magnetic beads (New England BioLabs, USA) at 4°C. Then, the beads were washed with PBS, and RNA was extracted through the Trizol reagent.

### Animal Assay

BALB/C nude mice (4 weeks old) were purchased from the Experimental Animal Center of Nanfang Hospital in Guangzhou, China for the following two experiments. Continuous dilutions of PANC-1 and AsPC-1 cell suspensions (5 105, 5 104, and 5 103 cells) with or without 5 106 MSCs were subcutaneously injected into nude mice in the first experiment. Six weeks later, the mice were killed, and tumor development was assessed. Furthermore, cells were transfected with pcDNA3.1/SNGH7 or pcDNA3.1, then, weekly intraperitoneally injected into mice treated with or without Folfirinox. Every 4 days, the volume of the tumors was determined. The mice were killed after 4 weeks, and the weight of the tumors was assessed.

### Transwell Assay

Normal pancreatic cancer cells were taken, digested, and centrifuged, and the supernatant was discarded and added to the RPMI-1640 basic medium. The cells were mixed and counted, placed in an empty 6-well plate, and placed in an incubator. The second to fifth generation of MSCs with normal growth were taken, digested, and centrifuged, and the cells were mixed with the complete medium of MSCs and counted. The final number of cells per well was about 5 x 105. Four hours later, the culture medium in the 6-well plate was sucked out and cleaned with PBS, replaced with the same amount of RPMI-1640 basic medium, and the cells were placed in the empty 6-well plate and placed in the incubator. After cleaning with PBS, each chamber was placed in a 6-well plate with 1.5 mL of methanol solution and fixed for 20 minutes. The chambers were dried, the cells on the surface of the compartment were wiped, and the cells were placed in a 6-well plate with 1.5 mL of crystal violet dye solution and stained in the dark for 60 minutes. The chambers were observed under a 200-magnification microscope.

### Statistical Analysis

Three different experiments yielded the following results. All of the results were provided as means standard deviations. GraphPad Prism 7.0 was used to analyze the data, which included one-way ANOVA and t-tests (GraphPad Inc., San Diego, CA, USA). P < 0.05 was regarded as statistically significant.

## Results

### MSCs Promote the Stemness and Folfirinox Resistance of Pancreatic Cancer Cells

**Figure 1:**
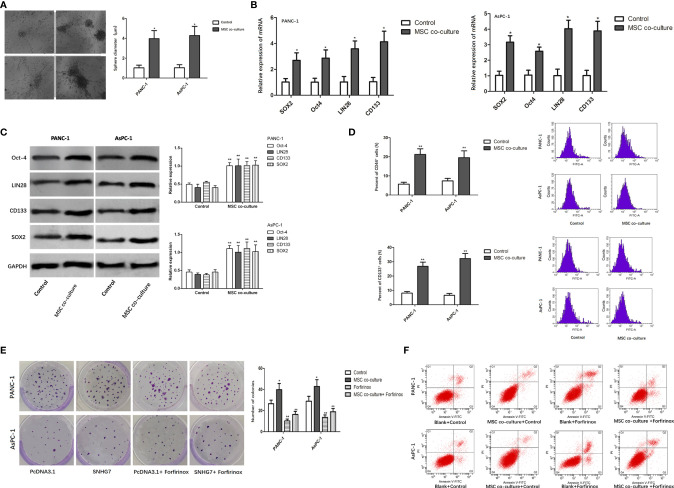
MSCs promote the stemness and Folfirinox resistance of pancreatic cancer cells. **(A)** Representative images and quantification of the tumor spheres generated by PANC-1, AsPC-1 cells with or without the co-culture with MSCs. **(B, C)** The expression of stemness markers were analyzed by using qRT-PCR and Western blot assays. **(D)** Flow cytometry analysis showed the rate of CD44+ cells upon MSC co-culture. **(E, F)** Colony-formation and flow cytometry analyses to detected the PANC-1, AsPC-1 cells with or without MSCs. *P < 0.05, **P < 0.01.

In order to determine whether MSCs can promote the stemness and Folfirinox resistance of pancreatic cancer cells, pancreatic cancer cell lines, PANC-1 and AsPC-1, were co-cultured with MSCs *via* a transwell co-culture system. Consequently, the ability of PANC-1 and AsPC-1 cells to generate tumor spheres was enhanced under the co-culture with MSC (**Figure 1A**). Furthermore, co-culture of MSCs greatly increased the expression of stemness genes such as SOX2, Oct-4, LIN28, and CD133 at both the mRNA and protein levels (**Figures 1B, C** **)**. According to the flow cytometry experiment, the number of CD44 positive (CD44+) pancreatic cancer cells, which are considered as cancer stem cell (CSC) characteristic indicators, was also enhanced (**Figure 1D**).

The findings of a colony formation experiment revealed that the growth inhibitory effects of Folfirinox resistance on pancreatic cancer cells were significantly reduced in the presence of MSCs (**Figure 1E**). Furthermore, MSC co-culture lowered GC cell apoptosis, and MSC co-culture inhibited the inductive impact of Folfirinox on pancreatic cancer cell apoptosis (**Figure 1F**). These findings indicated that MSCs can promote the stemness and Folfirinox resistance in pancreatic cancer cells.

### Effect of MSC in Xenograft Models

**Figure 2:**
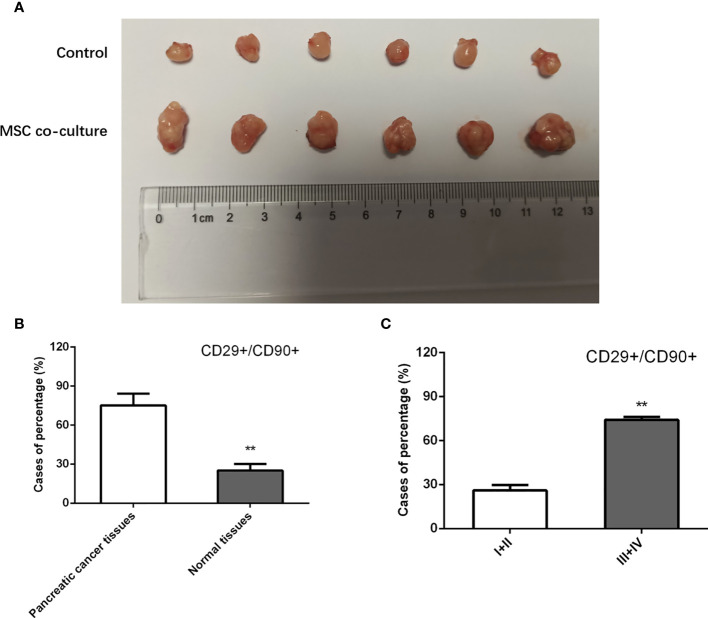
Effect of MSC in xenograft models. **(A)** Nude mice were injected with PANC-1 cells, and tumor weight in mice of each group were assessed. **(B, C)** Quantification of IHC staining rate of CD29+CD90+ in non-tumorous specimens or pancreatic cancer specimens from patients at stages I/II and III/IV. **P < 0.01.

To further investigate the effect of MSCs on tumor initiation, we subcutaneously injected a limiting dilution of PANC-1 cells at three dosages, 5 × 10^3^, 5 × 10^4^, and 5 × 10^5^, with or without 5 × 10^6^ admixed MSCs into nude mice. For 4 weeks, each group got Folfirinox intraperitoneal therapy once every 2 days. As a consequence, we discovered that PANC-1 cells alone at 5 103 and 5 104 cells failed to form xenografts. However, as compared to the injection of pancreatic cancer cells alone, cell combination with MSCs efficiently produced a xenograft and enhanced the weight of tumors transplanted, indicating that MSCs trigger pancreatic cancer cell *de novo* tumor development (**Figure 2A**). In addition, we used IHC labeling to identify the expression of MSC surface antigens, CD29 and CD90, in pancreatic cancer tissues. The results showed that the proportion of CD29+/CD90+ patients in pancreatic cancer tissues was substantially greater than in the responder group, and the rate of CD29+CD90+ in GC specimens was favorably connected with the clinical stage (**Figures 2B, C** **)**. MSCs boosted the stem characteristics and chemo-resistance in pancreatic cancer cells according to the findings.

### SNHG7 Is Induced by MSCs and Contributes to the Stemness and Folfirinox Resistance

**Figure 3:**
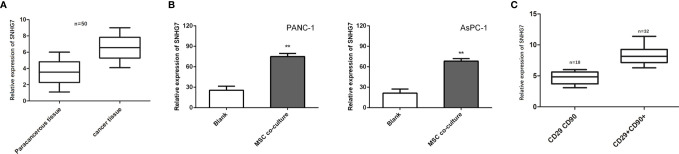
SNHG7 is induced by MSCs and contributes to the stemness and Folfirinox resistance. **(A, B)** SNHG7 expression was determined *via* qRT-PCR in pancreatic cancer tissues or cells with or without co-culture of MSCs. **(C)** The correlation between SNHG7 expression and co-expression of CD29 and CD90 in pancreatic cancer tissues. **P < 0.01.

Previous research has found that SNHG7 is overexpressed in pancreatic cancer tissues and promotes metabolic plasticity *via* antioxidant synthesis. As a result, we postulated that SNHG7 could have a role in the stemness and chemoresistance. SNHG7 was shown to be increased in pancreatic cancer tissues as compared to normal tissues (n = 50; **Figure 3A**). To identify whether SNHG7 was induced by MSCs, we detected its expression in PANC-1 and AsPC-1 cells co-cultured with or without MSCs. We found that the expression of SNHG7 was obviously increased both in pancreatic cancer after the co-culture with MSCs when compared to non-cultured cells (**Figure 3B**). In addition, we also performed *in situ* hybridization (ISH) to evaluate the correlation between SNHG7 expression and co-expression of CD29 and CD90 in pancreatic cancer tissues, which are well-known MSC surface antigen markers. We found that SNHG7 expression was higher in pancreatic cancer tissues that were both CD29 and CD90 positive (CD29+CD90+) than in pancreatic cancer tissues that were both CD29 and CD90 negative (CD29CD90) (**Figure 3C**). These findings suggest that SNHG7 may play a role in the effects of MSCs on pancreatic cancer cell stemness.

**Figure 4:**
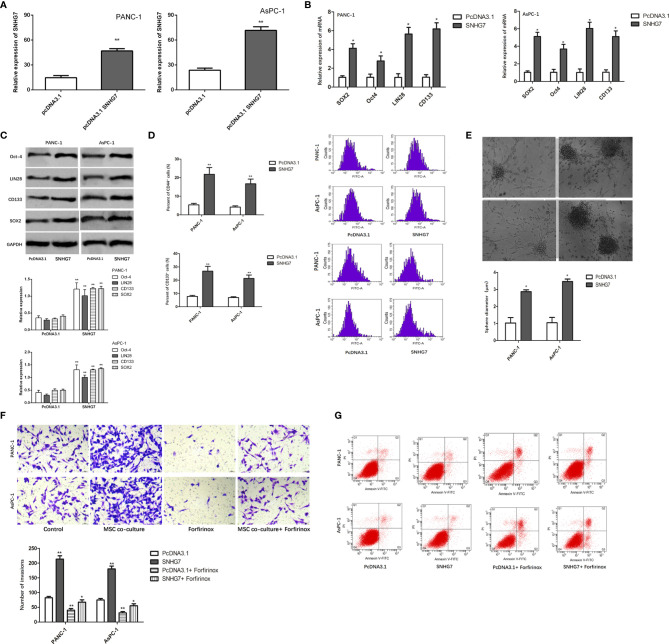
The effect of SNHG7 on the stemness and Folfirinox resistance. **(A, B)** overexpression efficiency was examined was verified by RT-qPCR and Western blot analyses. **(C)** Western blotting for stemness-associating genes in PANC-1 and AsPC-1 cell culture either alone or with MSCs and with or without Folfirinox. **(D)** The proportion of CD44^+^ pancreatic cancer cells. **(E)** Sphere-formation assay in PANC-1 and AsPC-1 cell culture either alone or with MSCs and with or without Folfirinox. **(F, G)** Colony-formation assay and flow cytometric analysis of PANC-1 and AsPC-1 cell culture either alone or with MSCs and with or without Folfirinox. *P < 0.05, **P < 0.01.

We investigated the effect of SNHG7 on the stemness and Folfirinox resistance. SNHG7 was overexpressed in stable lentivirus transfected way, and the overexpression efficiency that was examined was verified by RT-qPCR analyses (**Figure 4A**). The findings of the RT-qPCR and Western blot studies revealed that the expression of SNHG7 and stemness genes was significantly elevated in PANC-1 and AsPC-1 cells after transfection with pcDNA3.1 SNHG7 compared to the NC group (**Figures 4B, C** **)**. Flow cytometric analysis revealed that SNHG7 overexpression enhanced the fraction of CD44+ pancreatic cancer cells (**Figure 4D**). Besides, sphere-formation assay indicated that the sphere formation capability of PANC-1 and AsPC-1 cells was obviously increased after SNHG7 overexpression (**Figure 4E**). To evaluate whether SNHG7 is functionally involved in pancreatic cancer progression, transwell assay and flow cytometric analysis were performed. When treated with Folfirinox, the induced expression of SNHG7 promoted invasion and abolished growth inhibition, as demonstrated in **Figure 4F**. SNHG7 overexpression decreased cell apoptosis, and Folfirinox-induced apoptosis was reversed by SNHG7 ectopic expression in pancreatic cancer cells (**Figure 4G**). These results clearly indicated that MSC caused the stemness and Folfirinox resistance *via* inducing SNHG7.

### SNHG7 Is Interacted With Notch1 to Regulate the Stemness and Folfirinox Resistance in Pancreatic Cancer

**Figure 5:**
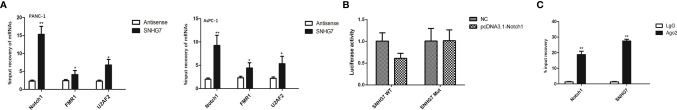
SNHG7 interacted with Notch1 to regulate the stemness and Folfirinox resistance in pancreatic cancer. **(A)** RT-qPCR western blot analyses revealed that three of the most upregulated miRNAs in pancreatic cancer cells respond to co-culture with MSC. **(B)** Pull down assay depicted that only Notch1 could be pulled down by SNHG7. **(C)** RIP analysis demonstrated the co-immunoprecipitation of SNHG7 and Notch1. *P < 0.05, **P < 0.01.

To further explore the regulatory mechanism of SNHG7, we searched for the target mRNAs for SNHG7. Prediction results from starBase3.0 (http://starbase.sysu.edu.cn/) showed that SNHG7 potentially interacted with miRNA. Using RT-qPCR, the three most substantially elevated mRNAs in pancreatic cancer cells in response to co-culture with MSCs were Notch1, FMR1, and U2AF2 (**Figure 5A**). In a pulldown test, SNHG7 could only pull down Notch1 rather than antisense SNHG7 in pancreatic cancer cells (**Figure 5B**), showing that SNHG7 interacted with Notch1. RIP assay further confirmed the direct interaction between SNHG7 and Notch1 (**Figure 5C**). Therefore, we deduced that Notch1 was a target for SNHG7 in pancreatic cancer.

**Figure 6:**
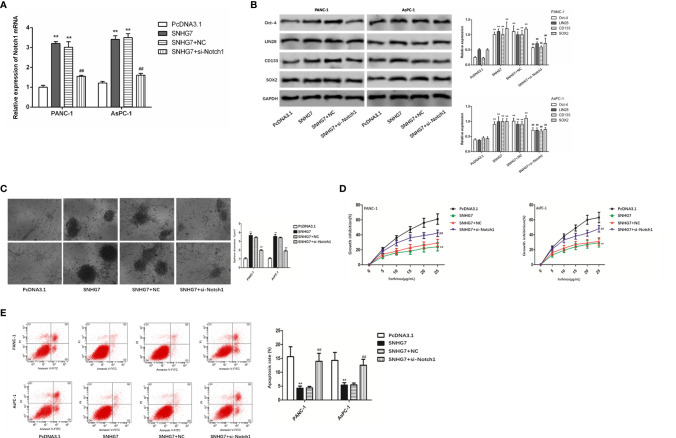
Notch1 to regulate the stemness and Folfirinox resistance in pancreatic cancer. **(A)** Notch1 expression was assessed *via* RT-qPCR assay. **(B)** The expression of stemness markers were analyzed by using Western blot assay. **(C–E)** Sphere-formation assay, Colony-formation, and flow cytometry analyses. **P < 0.01 (compared with pcDNA3.1), ^##^P < 0.01 (compared SNHG7+NC with SNHG7+si-Notch1).

Moreover, we tried to examine whether SNHG7 modulated stemness and resistance in pancreatic cancer cells through Notch1. RT-qPCR findings revealed that SNHG7 overexpression promoted Notch1 expression, but si-Notch1 co-transfection restored Notch1 expression levels in PANC-1 and AsPC-1 cells (**Figure 6A**). The co-transfection of si-Notch1 suppressed the protein levels of the stemness markers caused by SNHG7 overexpression (**Figure 6B**). Ability of PANC-1 and AsPC-1 cells to form tumor spheres was enhanced by SNHG7 overexpression and repressed by Notch1 downexpression (**Figure 6C**). CCK8 assay demonstrated that the inhibitive effect of Folfirinox on cell viability was reduced by SNHG7 overexpression and regained by the silenced expression of Notch1 (**Figure 6D**). Also, under the treatment of Folfirinox, apoptosis in PANC-1 and AsPC-1 cells was reduced by SNHG7 overexpression, and rescued by co-transfection of si-Notch1 (**Figure 6E**). Hence, our findings indicated that SNHG7 interacted with Notch1 to regulate the stemness and Folfirinox resistance in pancreatic cancer.

### MSC-Induced SNHG7 Facilitate Stemness and Folfirinox Resistance Through the Notch1/Jagged1/Hes-1 Signaling Pathway in Pancreatic Cancer

**Figure 7:**
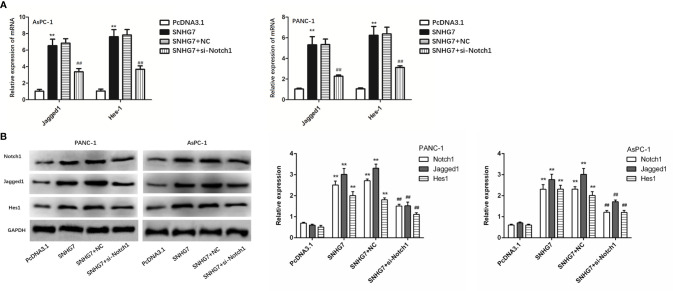
MSC-induced SNHG7 facilitate the stemness and Folfirinox resistance through the Notch1/Jagged1/Hes-1 signaling pathway in pancreatic cancer. **(A, B)** The expressions of the Notch1/Jagged1/Hes-1 signaling pathway related mRNA and proteins were determined by qRT-PCR and Western blot assays. **P < 0.01 (compared control with pcDNA3.1), ^##^P < 0.01 (compared SNHG7+NC with SNHG7+si-Notch1).

The expressions of the Notch1/Jagged1/Hes-1 signaling pathway components (Notch1, Jagged1, and Hes1) in PANC-1 and AsPC-1 cells were examined by qRT-PCR and Western blot tests to assess the biological relevance of SNHG7 in pancreatic cancer stemness and Folfirinox resistance. The results showed that the expressions of Notch1, Jagged1, and Hes1at mRNA and protein levels were significantly upregulated in PANC-1 and AsPC-1 cells with the transfection of pcDNA3.1 SNHG7 compared with the NC group (**Figures 7A, B** **)**.

## Discussion

Pancreatic cancer is a lethal solid tumor that usually causes local invasion and early spread, killing more than 300,000 individuals each year. PC patients have a poor prognosis, with less than 5% of patients living longer than 5 years (12). As a result, developing possible diagnostic markers or targeted medications to halt the progression of pancreatic cancer is important.

As an important component of the tumor environment, MSCs are a heterogeneous stroma/stem-like phenotype with the ability to differentiate along the mesodermal lineage (13). They may promote chemotherapy resistance by secreting protective cytokines or even generating genetic mutations and altering transcriptional expression to assist cancer cells in overcoming the anticancer effect of chemotherapeutic agents (14, 15). Recently, it has been demonstrated that MSCs play an important role in tumor chemoresistance (16). MSCs have been shown to release polyunsaturated fatty acids KHT and 16:4(n3), which can lead to resistance to platinum-based chemotherapy (17). Thus, it is unclear whether MSCs play a substantial role in metabolic reprogramming in the control of chemotherapy resistance.

As sequencing technology has advanced, more non-coding RNA has been identified. Among these, lncRNAs have gotten a lot of interest because of their diverse roles in all stages of carcinogenesis and tumor development, and lncRNAs might be exploited as novel prognostic indicators (18, 19). By influencing oncogenes or tumor suppressor genes, they contribute to tumor development, proliferation, and metastasis (20, 21). A growing body of data suggests that lncRNAs have a role in cancer stemness and treatment resistance (22, 23).

Small nucleolar RNA host gene 7 (SNHG7) has been shown to be carcinogenic in ovarian cancer (9). In this study, we looked at the unique biological effects of SNHG7 in pancreatic cancer. The key finding of this study is that SNHG7 plays a significant role in pancreatic cancer. We explored the critical function and post-transcriptional regulation of SNHG7 in MSC-induced stemness and Folfirinox resistance in this work. We found that the overexpression of SNHG7 confers Folfirinox resistance and enhances the stemness of pancreatic cancer cells.

Mechanistically, the interaction of lncRNAs with mRNAs in cancer progression and cellular response to chemotherapy has been widely documented. The Notch signaling system is an evolutionarily conserved system in the tumor microenvironment that is known to modulate the expression of its target genes and consequently plays an important role in various cellular processes such as cell proliferation, migration, and death (24). Notch receptors have been reported to be commonly dysregulated in several neoplastic lesions, demonstrating that Notch has an oncogenic function in a variety of malignancies (25). More crucially, mounting data suggests that the Notch signaling pathway is implicated in the chemoresistance of many tumor cells and also promotes radiation resistance in certain malignancies (26–28). We demonstrated that Notch1 interacted with SNHG7 and could reverse the facilitative effect of SNHG7 on the stemness and Folfirinox resistance in pancreatic cancer cells.

In conclusion, our findings show that MSCs increased SNHG7 expression in pancreatic cancer cells, promoting the stemness and Folfirinox resistance *via* the Notch1/Jagged1/Hes-1 signaling pathway. Our result not only helps us to better understand the regulatory potential of SNHG7 in pancreatic cancer stemness and Folfirinox resistance, but it is also important for identifying new pharmacological targets and devising innovative treatment techniques to overcome resistance.

## Data Availability Statement

The original contributions presented in the study are included in the article. Further inquiries can be directed to the corresponding authors.

## Author Contributions

All authors contributed to the article and approved the submitted version.

## Funding

This work was supported by the Interdisciplinary Program of Shanghai Jiao Tong University under grant No. YG2019QNB26.

## Conflict of Interest

The authors declare that the research was conducted in the absence of any commercial or financial relationships that could be construed as a potential conflict of interest.

## Publisher’s Note

All claims expressed in this article are solely those of the authors and do not necessarily represent those of their affiliated organizations, or those of the publisher, the editors and the reviewers. Any product that may be evaluated in this article, or claim that may be made by its manufacturer, is not guaranteed or endorsed by the publisher.
